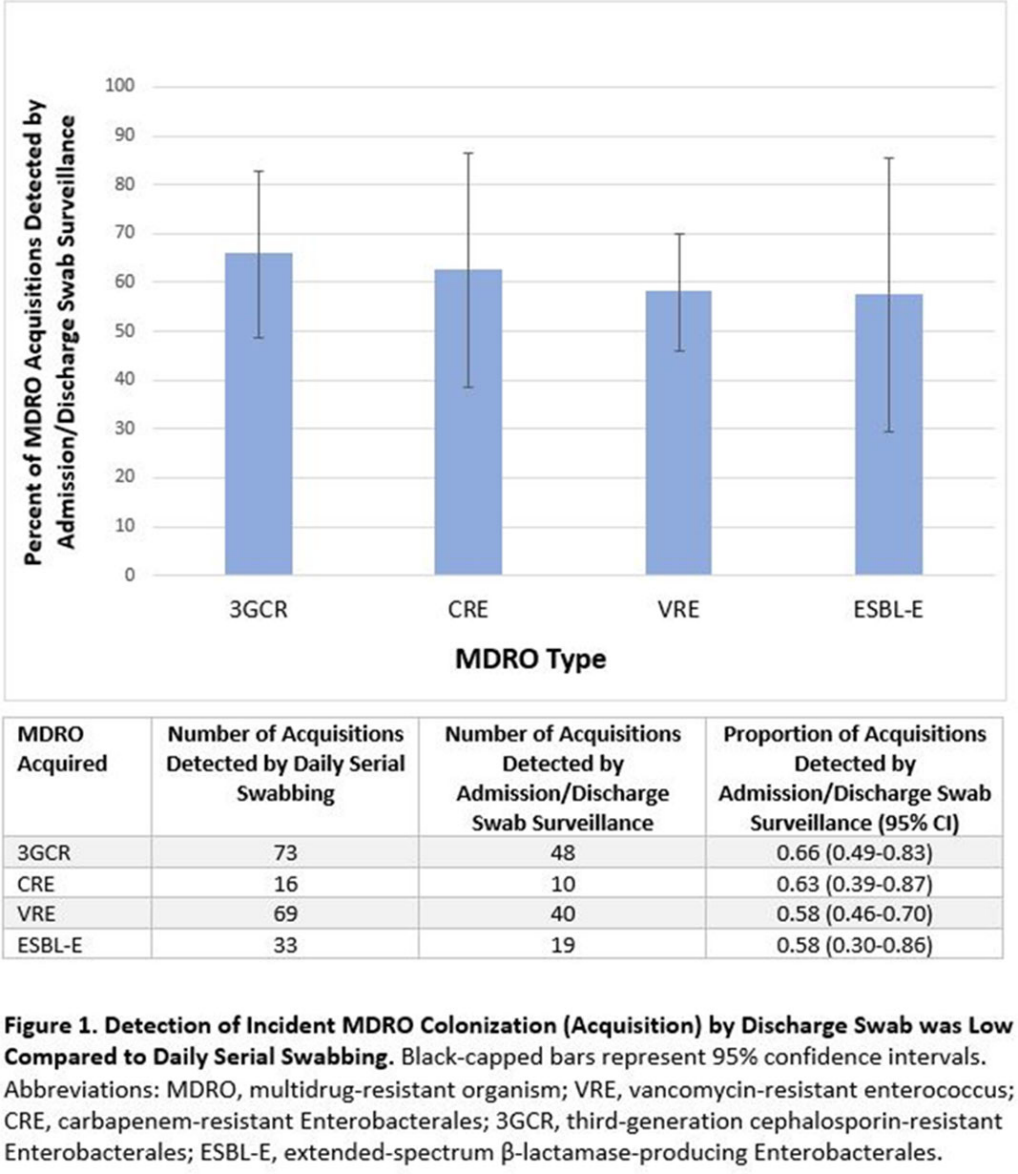# Admission and Discharge Sampling Underestimates Multidrug-Resistant Organism (MDRO) Acquisition in an Intensive Care Unit

**DOI:** 10.1017/ash.2021.51

**Published:** 2021-07-29

**Authors:** Sarah Sansom, Michael Lin, Christine Fukuda, Teppei Shimasaki, Thelma Dangana, Nicholas Moore, Rachel Yelin, Yoona Rhee, Lina Tabith, Jianrong Sheng, Enrique Cornejo Cisneros, John Murray, Kyle Chang, Karen Lolans, Michelle Ariston, William Rotunno, Hazel Ramos, Haiying Li, Khaled Aboushaala, Naomi Iwai, Christine Bassis, Vincent Young, Mary Hayden

## Abstract

**Background:** Identification of hospitalized patients with enteric multidrug-resistant organism (MDRO) carriage, combined with implementation of targeted infection control interventions, may help reduce MDRO transmission. However, the optimal surveillance approach has not been defined. We sought to determine whether daily serial rectal surveillance for MDROs detects more incident cases (acquisition) of MDRO colonization in medical intensive care unit (MICU) patients than admission and discharge surveillance alone. **Methods:** Prospective longitudinal observational single-center study from January 11, 2017, to January 11, 2018. Inclusion criteria were ≥3 consecutive MICU days and ≥2 rectal or stool swabs per MICU admission. Daily rectal or stool swabs were collected from patients and cultured for MDROs, including vancomycin-resistant *Enterococcus* (VRE), carbapenem-resistant Enterobacterales (CRE), third-generation cephalosporin-resistant Enterobacterales (3GCR), and extended-spectrum β-lactamase–producing Enterobacterales (ESBL-E) (as a subset of 3GCR). MDRO detection at any time during the MICU stay was used to calculate prevalent colonization. Incident colonization (acquisition) was defined as new detection of an MDRO after at least 1 prior negative swab. We then determined the proportion of prevalent and incident cases detected by daily testing that were also detected when only first swabs (admission) and last swabs (discharge) were tested. Data were analyzed using SAS version 9.4 software. **Results:** In total, 939 MICU stays of 842 patients were analyzed. Patient characteristics were median age 64 years (interquartile range [IQR], 51–74), median MICU length of stay 5 days (IQR, 3–8), median number of samples per admission 3 (IQR, 2–5), and median Charlson index 4 (IQR, 2–7). Prevalent colonization with any MDRO was detected by daily swabbing in 401 stays (42.7%). Compared to daily serial swabbing, an admission- and discharge-only approach detected ≥86% of MDRO cases (ie, overall prevalent MDRO colonization). Detection of incident MDRO colonization by an admission- or discharge-only approach would have detected fewer cases than daily swabbing (Figure 1); ≥34% of total MDRO acquisitions would have been missed. **Conclusions:** Testing patients upon admission and discharge to an MICU may fail to detect MDRO acquisition in more than one-third of patients, thereby reducing the effectiveness of MDRO control programs that are targeted against known MDRO carriers. The poor performance of a single discharge swab may be due to intermittent or low-level MDRO shedding, inadequate sampling, or transient MDRO colonization. Additional research is needed to determine the optimal surveillance approach of enteric MDRO carriage.

**Funding:** No

**Disclosures:** None

**Figure 1.**